# Increased weight-bearing load reduces biological body weight while sodium and water balances are unaffected

**DOI:** 10.1007/s00424-025-03114-3

**Published:** 2025-09-02

**Authors:** Jovana Zlatkovic, Jakob Bellman, Daniel Hägg, Mathilda Magnusson, Claes Ohlsson, Gerald DiBona, Fredrik Anesten, John-Olov Jansson

**Affiliations:** 1 Department of Physiology, Institute of Neuroscience and Physiology, Sahlgrenska Academy, University of Gothenburg, Medicinaregatan 11, 41390 Gothenburg, Sweden; 2 Sahlgrenska Osteoporosis Centre, Centre for Bone and Arthritis Research, Institute of Medicine, Sahlgrenska Academy, University of Gothenburg, 41345 Gothenburg, Sweden; 3 Department of Drug Treatment, Region Västra Götaland, Sahlgrenska University Hospital, 41345 Gothenburg, Sweden; 4 Departments of Internal Medicine and Molecular Physiology & Biophysics, Carver College of Medicine, University of Iowa, Iowa City, Iowa US-IA 52242 USA

**Keywords:** Obesity, Weight-bearing load, Sodium balance, Water balance, Body weight regulation

## Abstract

Increased weight-bearing load has previously been shown to reduce body weight in obese rodents, primarily by lowering food intake. However, it remains unclear whether increased loading also affects body weight through acute changes in extracellular water. This study aimed to determine whether increased weight-bearing load acutely produces negative sodium and water balances, as indicators of changes in whole-body extracellular sodium and water content. Diet-induced obese (DIO) rats were housed in metabolic cages with free access to hypotonic 0.5% NaCl solution and a low-sodium, high-fat diet. A novel, less traumatic loading method was used, where intra-abdominal capsules implanted two weeks earlier were either filled in vivo with wolfram granulate (Load group) or sham-filled (Control group), resulting in 15% and 2% increases in body weight, respectively. Compared to controls, increased weight-bearing load decreased body weight by 4.2% (95% CI -6.3, -2.0; *P* = 0.001) and reduced food intake by 1.6 percentage points (95% CI -2.6, -0.6; *P* = 0.003). No significant differences in sodium or water balance were observed. These findings suggest that load-induced reductions in body weight are not mediated by changes in whole-body extracellular sodium or water content, indicating that fluid homeostasis does not contribute to the homeostatic regulation of body weight.

**Supplementary Information:**

The online version contains supplementary material available at 10.1007/s00424-025-03114-3.

## Introduction

Worldwide prevalence of obesity has increased rapidly in recent decades and is now a global health concern that is associated with numerous comorbidities, such as cardiovascular disease (CVD), type 2 diabetes, and certain cancers [1, 25, 36]. Regulation of body weight is a complex process that involves the interplay of various physiological and environmental factors [21]. The connection between obesity and metabolic disorders is mainly believed to be caused by abnormal adipose tissue accumulation leading to dysfunctional adipose tissue which increases the risk for several comorbidities [11, 26]. Novel incretin-based treatments show great promise for the treatment of obesity and its comorbidities, but the underlying mechanism for body weight regulation is still not fully elucidated [29].

We have previously provided evidence for a new potential homeostatic regulator of body weight. This regulatory system acts by sensing changes in total weight-loading, leading to adjustments of body weight to maintain a set point. This proposed weight-detecting system has been named the gravitostat and has previously been shown to function independently of leptin and glucagon-like peptide-1 (GLP-1) [16, 22]. Increased loading has been shown to regulate body fat mass by influencing food intake and energy consumption [5, 23]. Moreover, we have demonstrated that increased load activates noradrenergic neurons in the Nucleus of the Solitary Tract (NTS), a brainstem area suggested as an integrating center for regulating energy homeostasis [35]. We have proposed that osteocytes in the weight-bearing bones can sense gravitational forces, i.e. weight-loading, and forward signals to the brain providing information about body weight status to help maintain body weight homeostasis [17]. Current methods of increasing weight-loading in rodents require surgical implantation of weight-capsules in the abdomen or subcutaneously (SC) on the lower back [16]. The abdominal surgery per se may cause weight loss due to the trauma and, thereby, confound the specific effect of weight-loading on body weight. Thus, it is important to develop new less traumatic loading techniques to determine the specific effect of increased weight-bearing load. Whether body weight changes caused by increased loading is affected by changes in other body compartments besides fat mass, such as total body water, is unknown.

Total body water is regulated by (1) water intake, which depends on factors triggering thirst; (2) by renal water excretion, which is regulated by several factors that affect the glomerular filtration rate (GFR) and the tubular reabsorption of water. Water and sodium balance are mainly regulated by feedback systems involving the hypothalamus, the neurohypophysis, the NTS, and the kidneys. These systems use osmosensing to influence thirst and renal tubular water reabsorption [12, 14, 19, 27, 28, 33]. Additionally, the extracellular fluid (ECF) determines the ECF osmolarity and sodium concentration. ECF regulation is mainly affected by changes in intravascular volume that trigger baroreceptors to alter sodium intake and/or excretion through various effectors [7, 8, 13, 20]. Therefore, ECF regulation relies mainly on information from a small portion of the ECF, the intravascular volume, and there is no known system that can detect the total amount of ECF or sodium in the body. It is possible that increased weight loading might exert part of its effect on body weight via regulation of body fluid levels. The aim of the present study was to determine if increased weight-loading, using a novel less traumatic loading method, affects body weight partly via influences on water and sodium balance.

## Methods

### Experimental design

**Fig. 1:**
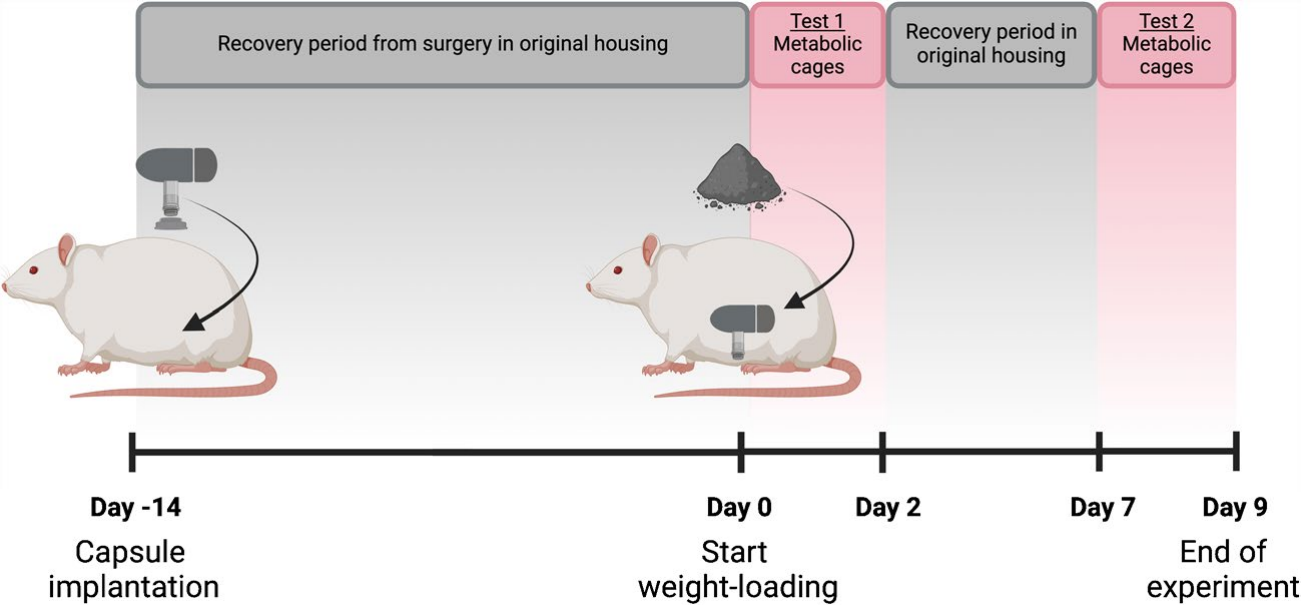
Schematic diagram of experimental protocol for the main study in metabolic cages. Diet induced obese (DIO) rats were intraperitoneally implanted with empty fillable capsules on Day −14. On Day −13 rats were put on low sodium high fat diet (LS-HFD) and saline drinking water. On Day 0 capsules were filled with wolfram granulate (Load) or left empty (Control) and the animals were put in metabolic cages for the first metabolic measurement (Test 1; Day 0 to 2). The second metabolic measurement (Test 2) took place between Day 7–9 after a five-day recovery period. Figure created with publication rights from BioRender.com

This experimental study used a novel less traumatic weight-loading method to determine how it affects body weight, food intake, water balance, and sodium balance in rats. The new weight-loading method was first evaluated in a pilot study to determine its efficacy. During this experiment, food was weighed daily to monitor food intake. The subsequent main metabolic study consisted of three phases over a little more than six weeks: (1) Day −35 to −15: induce obesity with diet, (2) Day −14 to −1: establish new sodium equilibrium by controlling sodium intake, (3) Day 0 to 9: add weight-loading and measure effect on body weight and food intake in addition to water and sodium balance in metabolic cages. During phase (2) empty fillable capsules were surgically implanted in the peritoneum, giving the animals a recovery period of two weeks before the start of phase (3). The weight-loading intervention during phase (3) lasted for a total of 9 days. The experimental data collection timeline is illustrated in Fig. 1. The local animal ethics committee approved all animal procedures (University of Gothenburg, Gothenburg, Sweden; Approval number 1874/18). All experiments were performed in accordance with relevant guidelines and regulations and are reported in agreement with ARRIVE (Animals in Research: Reporting In Vivo Experiments) guidelines.

### Animals

Male Sprague–Dawley rats, 10 weeks of age weighing approximately 350 g were purchased from Janvier Labs (Saint-Berthevin, France). Upon arrival, animals were group-housed under standardised conditions (reversed 12 h light/dark cycle [lights off at 11:00 a.m.], 50–60% humidity, 21 °C temperature). After a one-week acclimatisation-period in the animal facility, all rats were put on a high-fat diet ([HFD]; D12492: 60 kcal% fat, 0.13% sodium chloride; Research Diets, New Brunswick, NJ, USA). In the main metabolic study, rats remained on the HFD for three weeks, after which the diet was switched to a custom-made low sodium high-fat diet ([LS-HFD]; D21041201: 60 kcal% fat, 0.0081% sodium; Research Diets, New Brunswick, NJ, USA). This diet was maintained for the remainder of the study, approximately three additional weeks. At the time of the diet switch, animals were also given a 0.5% NaCl solution (saline water) that they consumed during the experiment. The LS-HFD and saline water were introduced two weeks prior to the start of the metabolic cage measurements to establish a new sodium equilibrium. By minimizing sodium content in the solid diet and delivering the majority of sodium through the drinking fluid, sodium intake could be quantified with higher accuracy. Throughout the experiment, animals had ad libitum access to plain water (sodium-free), saline water (0.5% NaCl), and either HFD or LS-HFD depending on the experimental phase.

### Weight-loading method

Rats were weight-matched and evenly divided into two groups (Control and Load). Sample size calculation was based on changes to body weight from similar previous studies [24, 35]. In total, two batches of animals with 20 rats (Load, n = 10; Control, n = 10) each were included. At approximately 14 weeks of age, corresponding to a continued growth phase, the rats were anaesthetised with isoflurane. The rat was placed on its back and an incision was made in the abdomen, followed by a cut of the peritoneum. An in-house manufactured fillable capsule (65 × 20 mm) was implanted intraperitoneally (IP). These hollow capsules, made from Sustarin C plastic, were shaped like hollow cylinders, with a catheter-like opening to the exterior, which enabled rapid filling in vivo. The fillable capsules were designed with a threaded tube (5 mm diameter) exiting from the capsule, through the skin to the exterior. The capsule was attached to the peritoneum with a nut, threaded on to the capsule-tube and placed between the peritoneum and the skin. On the skin surface the tube was sealed with a threaded cap enabling filling of the IP-placed capsule. The peritoneum was closed with sutures and the skin was closed with surgical staples. All rats were subcutaneously injected with analgesic (5 mg/mL Rimadyl; dose: 1 mL/kg, Orion Pharma Animal Health, Sollentuna, Sweden). The rats were allowed to recover for two weeks after surgery. Thereafter, the animals were briefly anaesthetised with isoflurane. During anaesthesia, the animals were placed on their backs and the nuts were removed. The capsules were either loaded with wolfram granulate (Edstraco AB, Rönninge, Sweden), equivalent to ~ 15% of their body weight (Load) or kept empty, weighing ~ 2% of their body weight (Control), to mimic an instant weight gain. All capsules were pre-weighed prior to implantation, with the empty capsule (including cap) weighing approximately 10.11 g. For the Load group, the final mass of the filled capsule was calculated to approximate 15% of the individual animal’s body weight. Investigators had to be aware of which capsules were loaded or kept empty during filling, therefore blinding was not feasible. The animals were included in the studies if they underwent a successful capsule implantation including no post-operative complications. Animals were excluded if there were signs of them feeling unwell (e.g. wound infection, deviant energy intake, or signs of lethargy) after surgery or if any capsule malfunction occurred.

### Metabolic cage trials

Three separate 48-h measurements were performed in metabolic cages (Tecniplast Group, Buguggiate, Italy): Baseline measurement (Measurement 0) at Day −7 to −5 (1 week after surgery), Measurement 1 on Day 0 to Day 2 (directly after loading), and Measurement 2 on Day 7 to 9 (1 week after loading). Baseline measurement was used as acclimatisation period for the animals to adapt to the metabolic cages. Additionally, the first 24 h of each 48-h measurement is considered equilibrium period and data from this period are not included in the analysis. For each measurement the animals were moved to individual metabolic cages and were kept under the aforementioned standardised housing conditions with ad libitum access to saline water and food (LS-HFD). The animals were put in metabolic cages in the morning directly before the lights turned off and were moved back to their original housing after 48 h. Body weight was measured at the start (0 h) and after 48 h. Food intake (gram [g]), water intake (millilitre [mL]), and urine volume (mL) were measured over a 24-h period at 24 h and 48 h. The food and water were pre-weighed before added to the cages and thereafter, at each measurement point, the amount left was weighed again. The urine was collected in specific containers filled with water-saturated mineral oil to avoid evaporation. Biological body weight was calculated by subtracting the capsule weight from the total body weight, accounting for both the empty capsules in the Control group and the filled capsules in the Load group. Changes in body weight are presented as percentage change in biological body weight relative to the start of loading (Day 0).

### Analysis of urine and sodium

Urine samples were stored at −20°C until analysis was performed. Electrolytes in urine were analysed with ion-selective electrodes by the Clinical Chemistry lab at the Sahlgrenska University Hospital (Gothenburg, Sweden) using standardised procedures with the Alinity analysis platform (Abbott, Illinois, USA). Sodium intake was calculated based on contributions from both drinking fluid and food. Total daily sodium intake was determined by summing the sodium content from these two sources. Sodium balance was then calculated as the difference between daily sodium intake and daily urinary sodium excretion.

### Statistical analysis

Data analysis was performed using SPSS Statistics 29 (IBM Corp., Armonk, NY, USA), Excel (Microsoft, Redmond, Washington, USA) and GraphPad Prism 10 (GraphPad Software, Boston, MA, USA). Figures were created with GraphPad Prism 10 (GraphPad Software, Boston, MA, USA) or BioRender.com (BioRender, Toronto, ON, Canada). Normality of the data was explored using Shapiro–Wilk test and Q-Q-plot (visual inspection) showing that presented data had normal distribution. The arithmetic mean values from each group (Load and Control) were calculated from the individual measurements and expressed with its corresponding standard error of the mean (mean ± SEM) or standard deviation (mean ± SD). All parameters were analysed using a two-way repeated measures analysis of variance (ANOVA), with group (Load vs Control) as the between-subjects factor and time as the within-subjects factor. The primary focus of the analysis was on the treatment × time interaction effect. When a significant interaction effect was observed, Bonferroni-corrected post hoc pairwise comparisons were conducted to identify specific time points with significant differences. Sphericity was not assumed; therefore, Greenhouse–Geisser–corrected degrees of freedom and p-values are reported values. A two-tailed, independent samples, Student's t-tests with equal variance assumption was used to evaluate baseline differences in body weight. *P*-values < 0.05 were considered statistically significant.

## Results

### Experimental animals

We determined if increased weight-loading regulates body weight in part by increasing sodium and water excretion, as an indication of a reduction of ECF. DIO rats had implanted capsules filled to weigh ~ 15% of their body weight (Load) or kept empty, weighing ~ 2% of their body weight (Control). Two batches of animals with 20 rats (Load, *n* = 10; Control, *n* = 10) each were used; one batch was used for the pilot study that tested the novel loading method and one batch was used for the main study in the metabolic cages. Some animals had to be excluded from the experiments and the data analysis due to complications. During the pilot study one rat in the Load group was removed due to surgical complications. During the main metabolic study, one rat was excluded from the Load group and two from the Control group due to surgical complications. Additionally, one rat was excluded from the control group due to capsule malfunction. None of the animals that were included in the final data analysis in the pilot study (Load, *n* = 9; Control, *n* = 10) or the main metabolic study (Load, *n* = 9; Control, *n* = 7) showed any signs of complications or adverse events during the experiments.

### Weight-loading reduces growth rate and food intake

First, in the pilot study, we evaluated the long-term effects (14 days) of the new loading method. A significant interaction between treatment and time was observed for the percentage change in biological body weight (*P* = 0.033; Supplemental Figure S1A). Post hoc analysis revealed that on day 2, the Load group exhibited a 1.3% lower increase in biological body weight compared to the Control group (95% CI −2.5, −0.1; *P* = 0.033; Supplemental Figure S1A). At the end of the experiment (day 14) the increase in biological body weight was 3.1% lower in the Load group compared to the Control group (95% CI −6.0, −0.2; *P* = 0.037; Supplemental Figure S1A). A significant interaction between treatment and time was also observed for the 48-h cumulative food intake across two consecutive days, expressed as percentage of biological body weight. Food intake was significantly lower in the Load group compared to the Control group throughout the long-term experiment (*P* = 0.003; Supplemental Figure S1B).

**Fig. 2:**
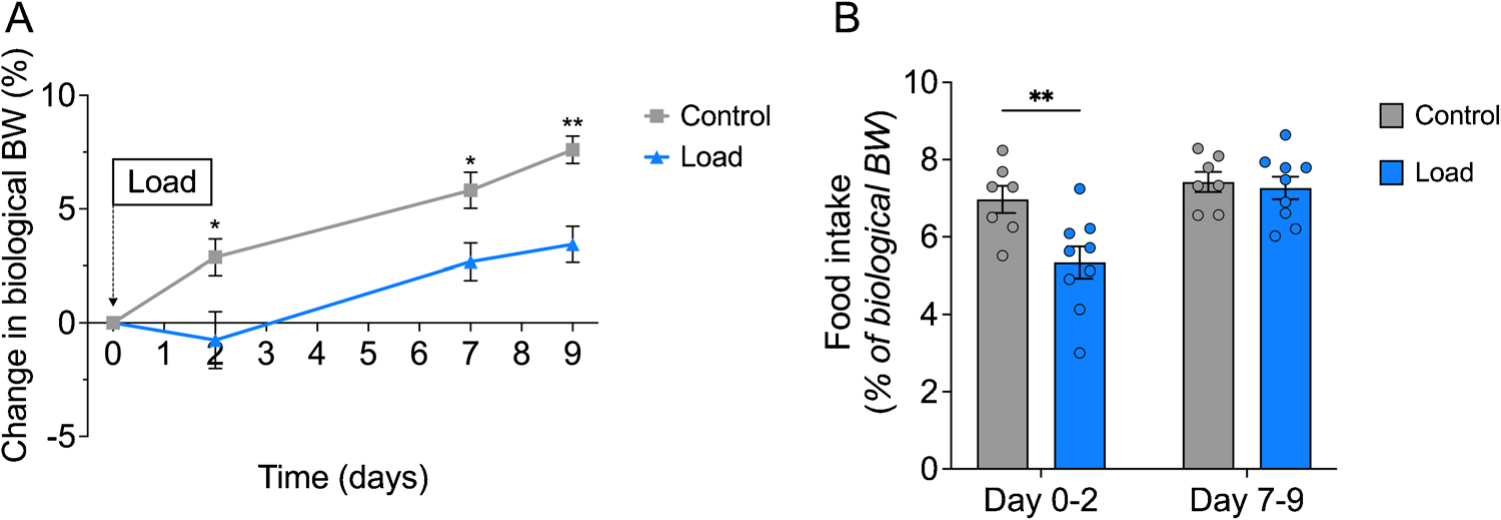
Weight-loading reduces weight gain and food intake in rats. Animals were treated with either high load (Load, *n* = 9) or low load (Control, *n* = 7). Measurements taken from two separate 48-h periods in metabolic cages: Measurement 1 (Day 0–2) and Measurement 2 (Day 7–9). **A** Percent change in biological body weight from start of loading, showing consistently reduced weight gain in the Load group compared to the Control group. **B** Cumulative food intake, expressed as percentage of biological body weight, was significantly lower in the Load group during Day 0–2, but not during Day 7–9. Interaction effects (treatment x time) were analysed using two-way repeated measures ANOVA, followed by Bonferroni-corrected post hoc comparisons. Data are expressed as mean ± SEM, with individual data points shown as circles. *p < 0.05, **p < 0.01

Next, in the main metabolic study, we performed a short-term (9-day) experiment investigating the effect of weight-loading on biological body weight, sodium balance and water balance. No significant baseline differences in body weight were observed between the groups (Supplemental Table S1). Repeated measures ANOVA revealed a significant interaction between treatment and time for body weight gain (*P* = 0.013; Fig. 2A). Post hoc analyses showed that two days after loading, the Load group exhibited a 3.6% lower increase in biological bodyweight compared to the Control group (95% CI −6.9, −0.4; *P* = 0.030; Fig. 2A). After nine days of loading, the difference increased, with the Load group showing a 4.2% lower weight gain compared to the Control group (95% CI −6.3, −2.0; *P* = 0.001; Fig. 2A). A similar pattern was observed when examining absolute changes in biological body weight, with the Load group demonstrating less weight gain than the Control group starting two days after loading (*P* = 0.018; Supplemental Figure S2).

A significant interaction between treatment and time was observed for the 48-h cumulative food intake from day 0 to 2, expressed as a percentage of biological body weight (*P* = 0.02; Fig. 2B). Food intake was 1.6 percentage points lower in the Load group compared to the Control group (95% CI −2.6, −0.6; *P* = 0.003; Fig. 2B), corresponding to a 20.0% reduction. Additionally, the cumulative absolute food intake was lower in the Load group compared to the Control group during Day 0 to Day 2 (Supplemental Figure S3).

### Weight-loading does not affect water or sodium balance

**Fig. 3:**
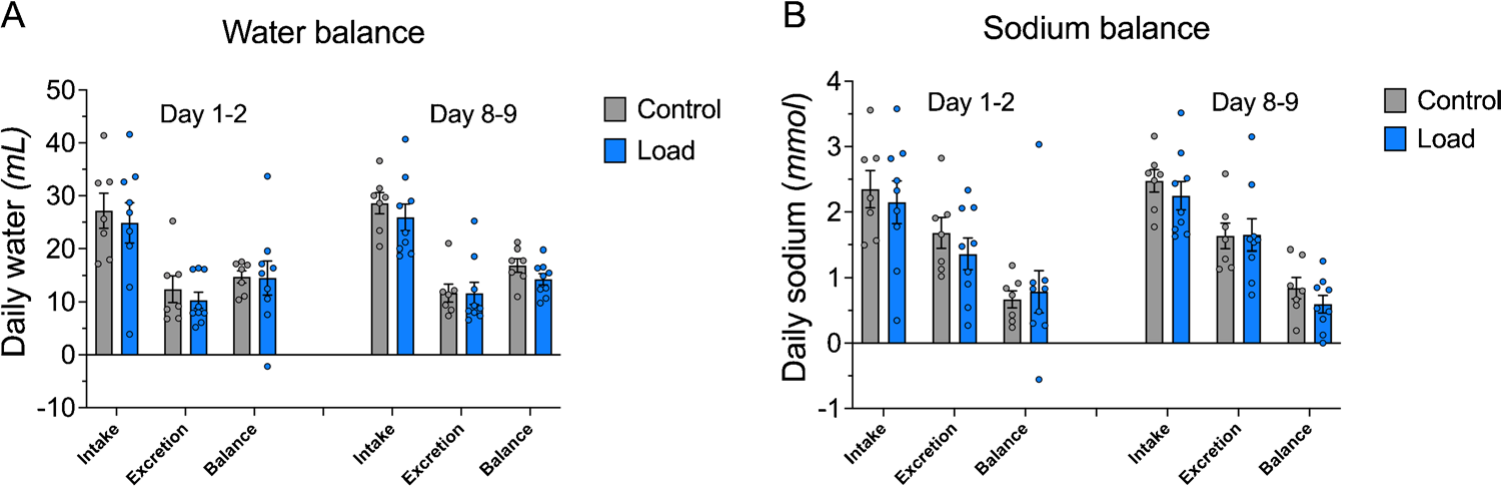
Weight-loading does not affect water or sodium balance. Animals were treated with either high load (Load, *n* = 9) or low load (Control, *n* = 7). Measurements were taken from 2 separate 48-h periods in metabolic cages: Measurement 1 (Day 0–2) and Measurement 2 (Day 7–9). Data from the final 24-h period of each measurement are presented. Total daily intake and excretion of water (mL) and sodium (mmol) were measured. Balance calculated as difference between intake and excretion. **A** No significant difference was observed between groups for water intake, water excretion or water balance. **B** Similarly, no significant differences were found for sodium intake, sodium excretion or sodium balance. Interaction effects (treatment x time) were analysed using two-way repeated measures ANOVA, followed by Bonferroni-corrected post hoc comparisons. Data are expressed as mean ± SEM, with individual data points shown as circles

To understand if the weight loss induced by loading could be explained by changes in water or sodium balance, two separate 48-h measurements in metabolic cages were performed during the short-term experiment. These measurements recorded the intake and output of sodium and fluids (water intake and urine excretion). Only data from the final 24-h period of each measurement (i.e. Day 1–2 and Day 7–9) are presented since the first 24-h period was considered equilibrium period. There was no difference between the Load and Control group in water or sodium intake, excretion, or balance during the first measurement on Day 1–2 (Fig. 3; Figure S4). Likewise, there was no difference between the groups in water or sodium intake, output, or balance during the second measurement on Day 8–9 (Fig. 3; Figure S4). In summary, increased weight-loading for nine days did not affect body water or sodium balance.

## Discussion

This study demonstrates that increased weight-bearing load reduces body weight gain in association with a reduction in food intake. Similar effects have previously been reported for IP and SC weight-bearing load when the load started at the time of capsule implantation [16, 17, 22]. In the present study, we introduce a novel, less traumatic two-step weight-loading method in which the load is added two weeks after capsule implantation. This enables the assessment of acute physiological responses directly after loading while minimizing potential effects from surgical trauma that may have confounded previous weight-loading methods. Using this method, we corroborate previous findings that increased loading reduces body weight gain [16, 22]. Importantly, the observed effect of increased loading on body weight was not associated with changes in sodium or water balance. We conclude that the effect on body weight is not due to load-induced effects on total body sodium content or total body water content.

The reduction in weight gain observed in this study is likely attributable to the decrease in food consumption after capsule filling. Previous studies have demonstrated that increased weight-loading mainly affects body weight by changes in fat mass while lean mass and bone mass remain unaffected [4, 16, 22]. Body composition was not evaluated, as our primary aim was to investigate acute changes in sodium and water balance. One potential concern is that intra-abdominal capsules might mechanically restrict gastric expansion and limit food intake. However, both groups received capsules of identical volume, differing only in filling weight. Furthermore, similar effects on body weight and food intake have been observed using subcutaneous capsules placed on the back, eliminating gastrointestinal interference [3, 35]. These observations indicate that the effect of weight-loading is physiological rather than a mechanical mechanism. Previous loading methods relied on surgically inserting capsules of different weight into the abdomen, resulting in an immediate increase in load [16]. This approach may introduce an acute confounding effect from the surgery that is difficult to distinguish from the specific effect of increased loading. Herein, we first implant empty capsules into the abdomen surgically. The animals recover for two weeks from the surgery, before adding the extra load by filling the capsules in vivo with wolfram granulate. This two-step procedure allows for more precise investigation of the physiological effects of increased loading without the confounding influence of surgery. Notably, the effects on body weight and food intake observed with this method are consistent with prior studies [4, 16, 22].

The lack of changes in water and sodium balance in the present study, both immediately after loading (Day 1–2) and one week later (Day 8–9), suggests that acute changes in extracellular fluid do not explain the load-induced reduction in growth rate. Instead, findings imply that the reduced growth rate is more likely driven by a decrease in energy intake rather than alterations in sodium and water excretion. Previous work has shown that weight-loading primarily affects body weight via reduced food intake, with no consistent changes in energy expenditure reported in rodents [16]. Although we did not measure energy expenditure, the additional workload from carrying the heavy capsules is likely to generate some increase in energy consumption, as observed in human studies [5, 24]. We speculate that weight-loading regulates body weight primarily by modulating fat mass through effects on energy intake, whereas sodium and water balance, along with ECF and total water, are regulated by other homeostatic systems [19, 30, 31, 34]. This is supported by our previous studies demonstrating that increased weight-loading reduces fat mass while lean body mass is unchanged [4, 16, 22].

Previous research by us and others has shown that the body weight reduction effect of increased weight-loading with weight capsules is more significant in obese mice on a high-fat diet compared to lean mice on a normal chow diet [2, 16, 32]. This observation suggests that the mechanism sensing weight-loading primarily protects against overweight since the effects are less pronounced in animals of normal weight [22]. One proposed mechanism for the reduced food intake and body weight due to loading is our earlier discovery that loading activates norepinephrine-producing neurons in the nucleus of the solitary tract (NTS), a brainstem area suggested to be an integrating center for meal termination and regulation of energy homeostasis [9, 15, 35]. Additionally, other areas within the NTS are known to be involved in the regulation of salt and water balance [18], providing a potential link between weight-loading and fluid homeostasis.

An advantage of the present study is the introduction of a minimally traumatic loading method. By filling the capsules after a recovery period, we avoided potential confounding effects of surgical trauma. Additional strengths include the use of identical metabolic cages for both groups, data collection performed by the same investigator, and consistent treatment of animals aside from the loading intervention. These measures likely minimized measurement variations, enhancing the reliability of between-group comparisons.

Several limitations should also be considered. First, although we assessed sodium and water balances, we did not measure extracellular or total body water content. Such measurements could have provided further insight into potential fluid shifts in response to loading. Techniques for quantifying total body water (e.g., isotope dilution or desiccation) or total body sodium (e.g., neutron activation analysis) were not feasible due to repeated use of animals, technical constraints, and lack of access to specialized equipment.

Second, while metabolic cages enabled precise measurement of intake and excretion, this setup can introduce stress-related physiological changes and may result in incomplete urine collection due to splashing or uneven distribution. A longer acclimatisation period might reduce these effects, but our 24-h period represented a compromise between minimizing stress over the extended protocol and adhering to ethical regulations. Additionally, brief anaesthesia was required for in vivo capsule filling, which may have introduced transient stress and potentially influenced physiological parameters. However, both groups underwent the same procedure, ensuring equal treatment and minimizing bias.

Third, although we observed positive sodium and water balances, these likely reflect a combination of unmeasured insensible losses (e.g., via skin, respiration, or faeces) and normal physiological variability. Faecal sodium excretion was not measured due to technical limitations. However, given the low sodium content of the diet and prior research indicating that faecal sodium accounts for only a small proportion of total intake even under higher dietary sodium conditions [10], the impact on our sodium balance interpretation is likely minimal.

Finally, we did not directly assess locomotor activity or energy expenditure. Prior studies using similar weight-loading paradigms have consistently shown reductions in body weight and food intake without clear changes in energy expenditure, suggesting that suppressed energy intake is a main driver [3, 6, 16, 35]. Despite these limitations, our findings consistently show that sodium and water balance remain unaffected by increased weight-loading, reinforcing the conclusion that fluid homeostasis is not a primary driver of the reduced growth rate. Future studies incorporating direct measurements of body fluid compartments, physical activity, and energy expenditure will help further elucidate the mechanisms of load-induced body weight regulation.

In conclusion, we present a less invasive weight-loading method that reduces body weight gain and food intake without affecting sodium or water balance. These findings suggest that load-induced body weight regulation is independent of acute changes in fluid homeostasis. Further studies are warranted to elucidate the underlying mechanisms of weight-loading and its role in body weight regulation.

## Supplementary Information

Below is the link to the electronic supplementary material.

Supplementary file1 (DOCX 3.58 MB)

## Data Availability

The data that support the findings of this study are available from the corresponding author upon reasonable request.
