# Supplementary material for: Increased weight-bearing load reduces biological body weight while sodium and water balances are unaffected
Source: Pflugers Arch. 2025 Sep 2;477(10):1287–94. doi: 10.1007/s00424-025-03114-3 (PMC12488816; doi:10.1007/s00424-025-03114-3)
Supplement: Supplementary file 1 — Supplementary file1 (DOCX 3.58 MB) [file 424_2025_3114_MOESM1_ESM.docx]

Supplementary Information

*Increased weight-bearing load reduces biological body weight while sodium and water balances are unaffected*

**Authors**

Jovana Zlatkovic, Jakob Bellman, Daniel Hägg, Mathilda Magnusson, Claes Ohlsson, Gerald DiBona, Fredrik Anesten, John-Olov Jansson

**Contents**

| **Figure S1** | Body weight and food intake from pilot study |
| --- | --- |
|  |  |
| **Figure S2** | Absolute changes in body weight in Load compared to Control |
|  |  |
| **Figure S3** | Cumulative absolute food intake from day 0-2 and day 7-9 in main metabolic study |
|  |  |
| **Figure S4** | Water and sodium balance normalised to biological body weight |
|  |  |
| **Table S1** | Baseline body weight before weight-loading intervention |
|  |  |

| **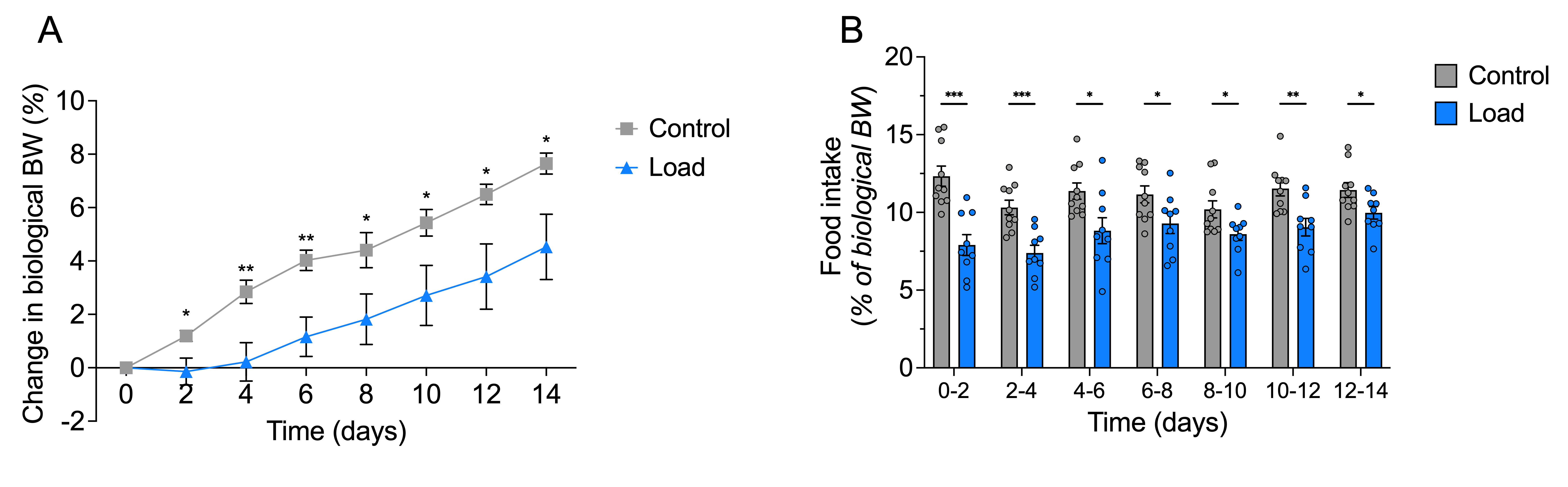**  **Figure S1: Long-term weight-loading reduces weight gain and food intake in rats.** Animals were treated with either high load (Load; n=9) or low load (Control; n=10) for 14 days using a novel method with fillable capsules implanted in the abdomen. **(A)** Percent change in biological body weight from the start of loading, showing reduced weight gain in the Load group compared to the Control group from Day 2 to Day 14. **(B)** Cumulative 48-hour food intake from two consecutive days, expressed as percentage of biological body weight, was consistently lower in the Load group compared to the Control group from Day 2 to Day 14. Interaction effects (treatment x time) were analysed using two-way repeated measures ANOVA, followed by Bonferroni-corrected post hoc comparisons. Data are expressed as mean ± SEM, with individual data points shown as circles. *p < 0.05, **p < 0.01, ***p < 0.001.  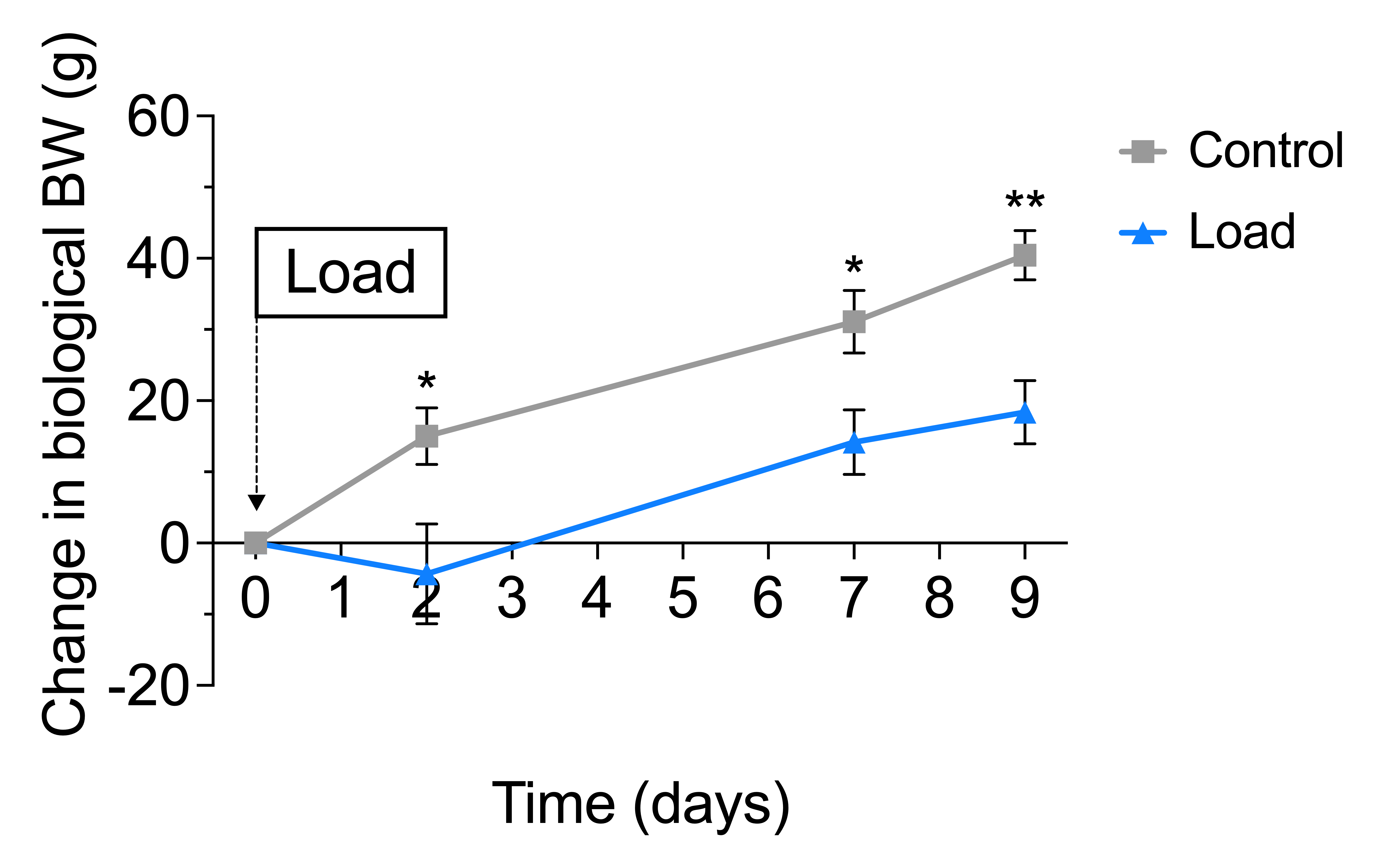  **Figure S2: Weight-loading reduces absolute weight gain in rats**. Animals were treated with either high load (Load, n=9) or low load (Control, n=7). Measurements were taken from two separate 48-hour periods in metabolic cages: Measurement 1 (Day 0-2) and Measurement 2 (Day 7-9). Absolute change in biological body weight from the start of loading, with consistently reduced weight gain observed in the Load group compared to the Control group across all time points. Interaction effects (treatment x time) were analysed using two-way repeated measures ANOVA, followed by Bonferroni-corrected post hoc comparisons. Data are expressed as mean ± SEM, with individual data points shown as circles. *p < 0.05, **p < 0.01.  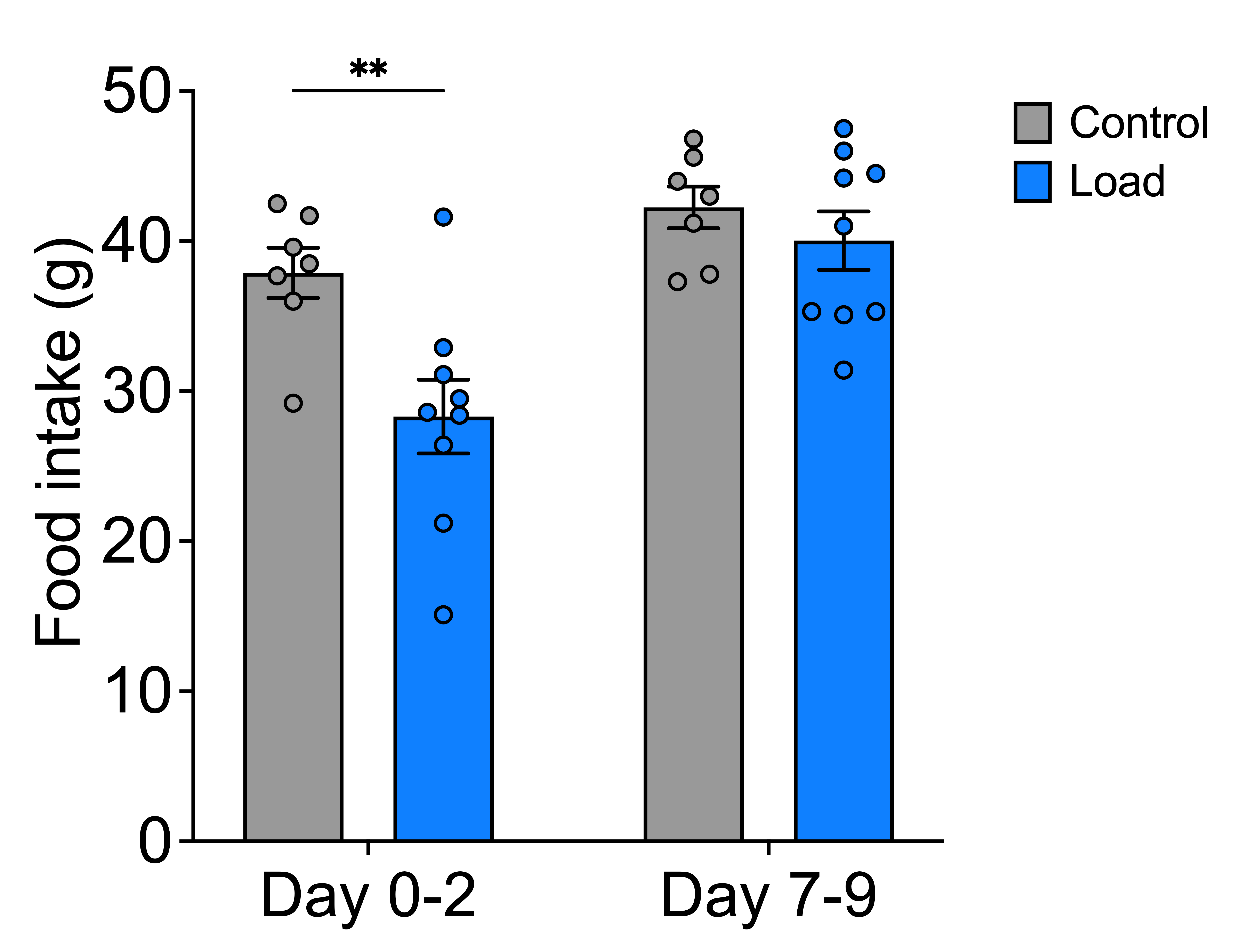  **Figure S3: Weight-loading reduces absolute food intake in rats.** Animals were treated with either high load (Load, n=9) or low load (Control, n=7). Measurements were taken from 2 separate 48-h periods in metabolic cages: Measurement 1 (Day 0-2) and Measurement 2 (Day 7-9). Food intake was significantly lower in the Load group during Day 0-2, but no difference was observed during Day 7-9. Interaction effects (treatment x time) were analysed using two-way repeated measures ANOVA, followed by Bonferroni-corrected post hoc comparisons. Data are expressed as mean ± SEM, with individual data points shown as circles. **p < 0.01. |  |
| --- | --- |

**
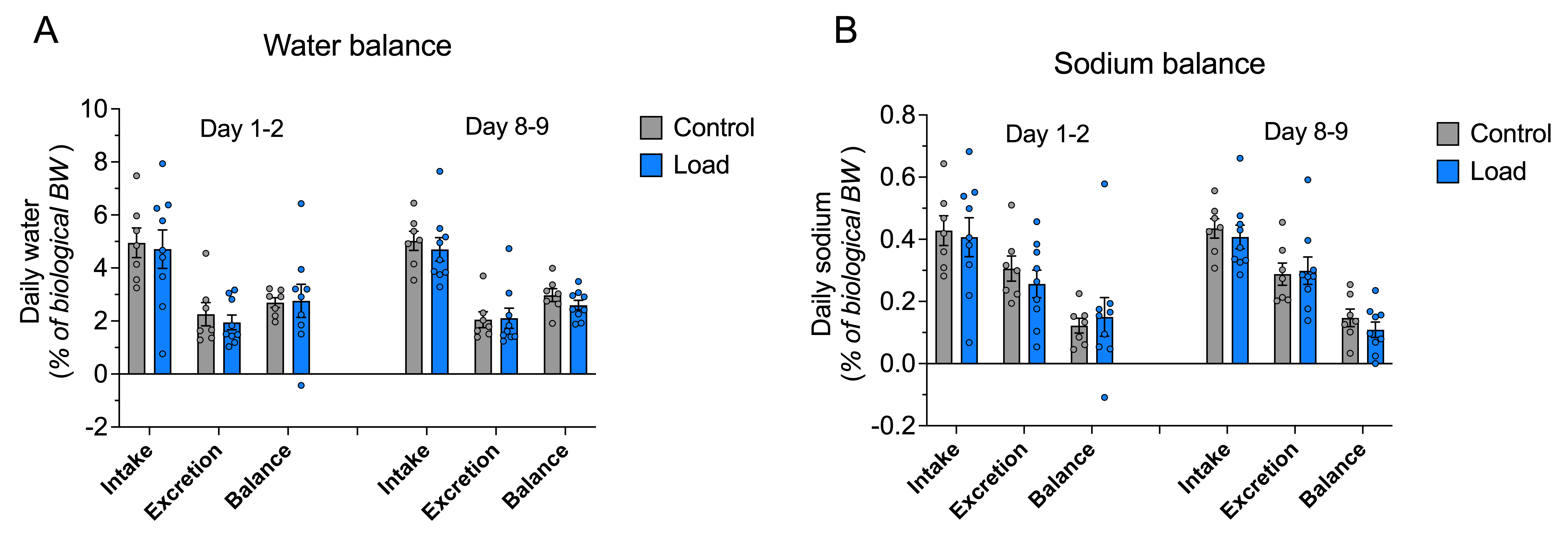
**

**Figure S4: Weight-loading does not affect water or sodium balance.** Animals were treated with either high load (Load, n=9) or low load (Control, n=7). Measurements were taken from two separate 48-hour periods in metabolic cages: Measurement 1 (Day 0-2) and Measurement 2 (Day 7-9). Data from the final 24-hour period of each measurement are presented. Total daily intake and excretion of water (mL) and sodium (mmol) were measured. Balance was calculated as the difference between intake and excretion, and values were normalised to body weight. **(A)** No significant differences were observed between groups for water intake, water excretion, or water balance. **(B)** Similarly, no significant differences were found for sodium intake, sodium excretion, or sodium balance. Interaction effects (treatment x time) were analysed using two-way repeated measures ANOVA, followed by Bonferroni-corrected post hoc comparisons. Data are expressed as mean ± SEM, with individual data points shown as circles.

**Table S1. Absolute biological body weights**

|  |  |  |
| --- | --- | --- |
| **Characteristics** |  |  |
|  | **Control** | **Load** |
|  | (n=7) | (n=9) |
| *Body weight (g)* |  |  |
| Day 0 | 530.1 ± 31.9 | 531.8 ± 24.3 |
| Day 2 | 545.1 ± 31.4 | 527.4 ± 25.7 |
| Day 7 | 561.2 ± 39.2 | 545.9 ± 27.5 |
| Day 9 | 570.5 ± 37.3 | 550.1 ± 30.5 |
|  |  |  |

**Table S1**. Values are presented as mean ± SD for all included animals. A Student's t-test indicated no significant baseline (Day 0) differences between high load (Load) and low load (Control) groups regarding body weight.
